# Philosophy in Homœopathy

**Published:** 1890-08

**Authors:** 


### BOOK NOTICES.

PHILOSOPHY IN HOMŒOPATHY. By Charles S. Mack, M. D., Professor of Materia Medica and Therapeutics in the Homœopathic Medical College of the University of Michigan, at Ann Arbor. Chicago, 48 Madison Street: Gross & Delbridge, 1890.

This clever little book of 174 pages consists of a series of essays upon Homœopathy as a law of practice. The writer stoutly maintains that Homœopathy is *a law* and not a variable rule of practice. His argument upon this ground is very well sustained and shows that he has thought long and deeply upon the question.

Nevertheless, we regret to have to confess he justifies the use of methods of practice which are not allowable or consistent under the homœopathic law; as, for instance, Quinine in massive doses for the treatment of intermittents; Opium and its alkaloid, Morphine, for the "relief" of pain, and Atropine injected into the eye in cases of iritis to roll up the iris and prevent adhesions. Thus the author, notwithstanding his forcible argument for the application of *the law*, opens wide the door of excuses that will permit the doubting practitioner to exercise his caprices or to gratify his prejudices by the use of methods of practice which we commonly call eclectic or mongrel, and then to screen himself from any consequences or any criticism of his folly by the plea of exercising his judgment or his reason.

Thus the conscientious adherence to a natural law, and deep trust in its efficacy, and the painstaking and relentless search for the simillimum, all may be set aside, and the incompetent physician, conspicuously wanting in these qualities of mind so needful for the successful homœopathist, may substitute for them the Imperial pride of intellect that sets itself up in disapproving "judgment" of the discoveries of transcendent genius, and sets aside as useless a plan of treatment which is really beyond its capacity to understand or to use. He therefore resorts to the well-known measures of the old school, to the damage of his patient and the shame of Homœopathy, while with impudent sophistry he defies the protest of the consistent Hahnemannian.

This permission, if we may call it so, thus given for these outside methods of practice, by reason of their bringing about the results in the foregoing statements, constitute a fault in the book that seriously impairs its usefulness.

Could this fault be eliminated (and it could be without mutilating the plan of the work), it would then be a very valuable means of instruction in the homœopathic principle. W. M. J.

